# Utilization of macrocyclic peptides to target protein-protein interactions in cancer

**DOI:** 10.3389/fonc.2022.992171

**Published:** 2022-11-17

**Authors:** Jiawen Yang, Qiaoliang Zhu, Yifan Wu, Xiaojuan Qu, Haixia Liu, Biao Jiang, Di Ge, Xiaoling Song

**Affiliations:** ^1^ Department of Thoracic Surgery, Zhongshan Hospital, Fudan University, Shanghai, China; ^2^ Shanghai Institute for Advanced Immunochemical Studies, ShanghaiTech University, Shanghai, China; ^3^ Shanghai Clinical Research and Trial Center, Shanghai, China; ^4^ School of Life Science and Technology, ShanghaiTech University, Shanghai, China; ^5^ University of Chinese Academy of Sciences, Beijing, China; ^6^ School of Physical Science and Technology, ShanghaiTech University, Shanghai, China; ^7^ CAS Key Laboratory of Synthetic Chemistry of Natural Substances, Shanghai Institute of Organic Chemistry, Chinese Academy of Sciences, Shanghai, China

**Keywords:** protein-protein interactions, macrocyclic peptide, cancer, treatment, drug

## Abstract

Protein-protein interactions (PPIs) play vital roles in normal cellular processes. Dysregulated PPIs are involved in the process of various diseases, including cancer. Thus, these PPIs may serve as potential therapeutic targets in cancer treatment. However, despite rapid advances in small-molecule drugs and biologics, it is still hard to target PPIs, especially for those intracellular PPIs. Macrocyclic peptides have gained growing attention for their therapeutic properties in targeting dysregulated PPIs. Macrocyclic peptides have some unique features, such as moderate sizes, high selectivity, and high binding affinities, which make them good drug candidates. In addition, some oncology macrocyclic peptide drugs have been approved by the US Food and Drug Administration (FDA) for clinical use. Here, we reviewed the recent development of macrocyclic peptides in cancer treatment. The opportunities and challenges were also discussed to inspire new perspectives.

## Introduction

Protein-protein interactions (PPIs) are the centers of most cellular processes. It has been proven that the dysregulation of PPIs can lead to the pathogenesis of various diseases, including cancer (1). One famous example is the negative regulation of tumor suppressor protein p53 by mouse double minute 2 (MDM2) and its homolog MDMX. Disruption of these PPIs exerts oncogenic activity (2). Another famous example is the interaction between programmed cell death protein 1 (PD-1) and programmed cell death ligand 1 (PD-L1), which plays a critical role in attenuating the immune response to cancer cells, thus leading to cancer immune escape (3, 4). Thus, targeting pathologic PPIs has gained more attention as an attractive strategy for cancer therapy.

Macrocyclic peptides have emerged as a class of ideal drug candidates to target PPIs. They are composed of various groups of molecules with a macrocyclic scaffold spanning from 5 to 14 amino acid residues, and their molecular weight is around 500-2000 (5, 6). As a structurally diverse class of molecules, macrocyclic peptides contain different types of molecules, including natural macrocyclic peptides, peptidomimetics, stapled peptides, β-hairpin mimetics, bicyclic peptides, and some macrocyclic peptides with unnamed structures (7, 8). Stapled peptides refer to cyclic peptides with α-helical conformations. Similarly, β-hairpin mimetics refer to cyclic peptides with β-hairpin motifs. Both α-helices and β-hairpins can locate at the active sites of various PPIs, and the conformations are the main determinants of the bioavailability and the bioactivity of such peptides (9, 10). Thus, stapled peptides and β-hairpin mimetics are ideal drug candidates. On the other hand, bicyclic peptides and other peptides with unnamed structures have also been developed to serve as novel drug candidates for disease therapy. And bicyclic peptides are a kind of peptides with two macrocyclic rings that allow bicyclic peptides to be bifunctional (11). All four types of macrocyclic peptides were used in drug development.

The diverse conformational space of macrocyclic peptides has captivated the imagination of medicinal chemists. With rapid advances in peptide drug discovery, there is a robust increase in the number of macrocyclic peptide antitumor agents having undergone or completed the early phase of clinical trials (12, 13). The details of those compounds tested in clinical trials including indications and targets were in **Table 1**. Furthermore, the FDA has approved two macrocyclic peptide drugs, pasireotide (2014) and lanreotide (2014), to treat patients with Cushing’s disease, acromegaly, and neuroendocrine tumors (**Table 2**) (13, 14). And these two drugs are derivatives of human hormones. To date, several platforms have been used to develop macrocyclic peptide drugs, including phage/mRNA display, splitintein circular ligation of peptides and proteins (SICLOPPS), one-bead one-compound (OBOC) libraries, and the random nonstandard peptides integrated discovery (RaPID) system. These prolific technologies enable researchers to generate specific and potent macrocyclic peptides against almost any protein target. The details of these technologies have already been well summarized in several previous reviews (5, 8, 15).

**Table 1:** Details of oncology macrocyclic peptide drugs in clinical trials.

Name	Indications(Phase, NCT number) ^a^	Molecular target	Target location	Discovery platform	Molecular Weight	Company
Cilengitide	Glioblastoma (phase III, NCT00689221) NSCLC (phase II, NCT00842712) Squamous cell carcinoma of the head and neck (phase II, NCT00705016) Melanoma (phase II, NCT00082875) Prostate cancer (phase II, NCT00103337) Leukemia (phase II, NCT00089388)	the integrins αvβ3 and αvβ5	Extracellular PPIs	Designed based on the RGD motif and spatial screening	588	Merck-Serono
Balixafortide	Breast cancer (phase III, NCT03786094) MM (phase 2, NCT01105403) Leukemia (phase II, NCT01413568)	CXCR4	Extracellular PPIs	Protein epitope mimetic	1864	Polyphor Ltd.
LY2510924	Leukemia (phase II, NCT02642871) Renal cell carcinoma (phase II, NCT01391130) Small cell lung carcinoma (phase II, NCT01439568)	CXCR4	Extracellular PPIs	Medium throughput screen and rational design	1189	Eli Lilly and Company
Motixafortide (BL-8040)	MM (phase III, NCT03246529) Pancreatic adenocarcinoma (phase II, NCT02826486) Gastric adenocarcinoma (phase I, II, NCT03281369) Leukemia (phase II, NCT02763384)	CXCR4	Extracellular PPIs	Downsized and modify-ed from a natural protein named T22	2159	BioLineRx Ltd.
ALRN-6924	solid tumor and lymphoma (phase II, NCT2264613) lung cancer (phase I, NCT04022876)	MDMX/MDM2	Intracellular PPIs	Phage display and further modifications	1930	Aileron Therapeutics, Inc.

a For drugs tested for different indications, the most advanced phase and latest trial are listed.

MDM, mouse double minute; PPI, protein-protein interaction; NSCLC, non-small cell lung cancer; RGD, Arg-Gly-Asp; MM, Multiple Myeloma; CXCR, C-X-C chemokine receptor.

**Table 2:** Details of oncology macrocyclic peptide drugs approved by the FDA (2008-2022).

Name	Indication	Molecular target	Target location	Source	Molecular Weight	Company
Lanreotide	Cushing’s disease, acromegaly, neuroendocrine tumors	Somatostatin receptor	Extracellular PPIs	Somatostatin analogs	1096	Ipsen
Pasireotide	Cushing’s disease, acromegaly	Somatostatin receptor	Extracellular PPIs	Somatostatin analogs	1047	Recordati Inc.

PPIs: protein-protein interactions.

In this manuscript, we reviewed the progress of macrocyclic peptide drugs focusing on their application in cancer treatment. First, we compared the benefits of using macrocyclic peptides to target protein-protein interactions instead of other drug candidates, such as small-molecule drugs and biologics. Then, we reviewed the progress of two types of oncology macrocyclic peptides separately based on the locations of their targets: extracellular or intracellular PPIs. Finally, we discussed the opportunities and challenges in discovering new macrocyclic peptide drugs.

## The advantages of using macrocyclic peptides to target PPIs

There are several advantages of using macrocyclic peptides to target PPIs over small-molecule drugs, linear peptides, and biologics [proteins and monoclonal antibodies (mAbs)] (**Table 3**). First, it can target protein PPIs interfaces generally ‘undruggable’ to both small-molecule drugs and biologics. It is difficult for small molecules to modulate PPIs because of a lack of binding pockets, poor selectivity, or small binding-surface area. However, peptides can interact with PPIs at multiple and distant sites with higher selectivity due to larger surface area and higher structural complexity (12). Biologics, on the other hand, are restricted to target extracellular PPIs because of poor cell permeability. Macrocyclic peptides can target extracellular and intracellular PPIs with relatively better cell permeability and tissue penetration. Second, it was estimated that the overall expense of peptide drugs might be lower than that of small-molecule drugs with the progression in synthetic methodology (16). Third, peptides have less immunogenicity, toxicity, and reduced off-target effects (5, 8). Furthermore, in comparison to linear peptides, cyclization not only can decrease polar surface area, increase cell permeability, and elevate stability to protease enzymes but also can enable peptides to interact with targeting proteins with less entropic binding cost in the correct three-dimensional conformation (17). Thus, macrocyclic peptides have occupied a specific space in targeting PPIs and attracted attention in recent years (**Table 4**).

**Table 3:** The difference among three major classes of therapeutic molecules.

Properties	Small molecules	Peptides		Biologics
		Linear	Macrocyclic	
Molecular weight	<500	500-2000		>5000
Binding surface area (Å^2^)	300-1000	1500-3000		1000-3000
Protease resistance	High	Moderate	High	Low
Cell permeability	High	Low	Moderate	Inability
Affinity for PPIs	Low	Moderate	High	High
Optimal targets	Enzymes	Intracellular/extracellular PPIs		Extracellular PPIs
Oral bioavailability	High	Low to moderate		Inability
Subtype selectivity	Low to moderate	Moderate to high		High
Representative drug	Crizotinib	Degarelix	Lanreotide	Nivolumab

PPIs: protein-protein interactions.

**Table 4:** Examples of macrocyclic peptides.

Compound no. and name	Target (Kd or EC50 or IC50 value for target)	Biological activity	Refs
1. Lanreotide	Somatostatin receptors (IC_50 =_ 57 nM in GH cells)	Approved to treat Cushing’s disease, acromegaly, and neuroendocrine tumors; *in vitro* (antitumor effect in oral squamous cell carcinoma cell lines)	(18, 19)
2. pasireotide	Somatostatin receptors (IC_50 =_ 0.4 ± 0.1 nM in rat pituitary cells)	Approved to treat Cushing’s disease and acromegaly; *in vitro* (antitumor effect in multiple thyroid cancer, prostate cancer and oral squamous cell carcinoma cell lines); *in vivo* (reduced metastasis in ductal pancreatic adenocarcinoma and pancreatic mouse xenograft models and antitumor effect in thyroid cancer mouse xenograft models)	(19–26)
3. Motixafortide	CXCR4 (IC_50 =_ 1 nM)	*In vitro* (induction of CXCR4-dependent cell death in leukemia and MM cell lines); *in vivo* (antitumor effect in leukemia, MM, NSCLC mouse xenograft models); clinical trials (NCT02826486, NCT01838395, and NCT02073019)	(27–34)
4. Balixafortide	CXCR4 (IC_50_<10 nM)	*In vitro* (inhibition of pERK/pAKT signaling in Namalwa and Jurkat cell lines and SDF-1 dependent chemotaxis in MDA-MB-231, Namalwa, and Jurkat cell lines); clinical trials (NCT01837095)	(35, 36)
5. LY2510924	CXCR4 (IC_50 =_ 0.0797 nM and Ki= 0.0495 nM in CCRF-CEM cells)	*In vitro* (inhibition of SDR-1- and CXCR4-mediated signaling pathways in HeLa cells); *in vivo* (antitumor effect in human NHL and multiple solid tumor xenograft models)	(37)
6. Peptide R54	CXCR4 (IC_50 =_ 1.5± 0.5 nM in CCRF-CEM cells)	*In vivo* (antiproliferative effect in a PES43 mouse xenograft model)	(38)
7. BMSpep-57	PD-L1(IC_50 =_ 9 nM and EC_50 =_ 566± 122 nM in Jurkat cells)	Untested *in vitro*	(39)
8. BMSpep-71	PD-L1(IC_50 =_ 7 nM and EC_50 =_ 293± 93 nM in Jurkat cells)	Untested *in vitro*	(39)
9. BMSpep-99	PD-L1(IC_50 =_ 153 nM and EC_50 =_ 6.3± 3.28 μM in Jurkat cells)	Untested *in vitro*	(39)
10. BMS-986189	PD-L1 (IC_50 =_ 1.03 nM)	Untested *in vitro*	(40)
11. C8	PD-1 (Kd= 0.64± 0.19 μM)	*In vitro* (activated CD8^+^ and CD4^+^ T cells); *in vivo* (antitumor effect in the CT26 mouse xenograft model)	(41)
12. D4-2	SIRPα (IC_50 =_ 0.18 μM)	*In vitro* (improved the Ab-dependent cellular phagocytosis activity of macrophages); *in vivo* (antitumor effect in Raji and B16BL6 mouse xenograft models)	(42)
13. HL2-m5	Sonic hedgehog (Shh) protein (Kd= 170± 20 nM and IC_50 =_ 250 nM)	*In vitro* (inhibition of Shh-dependent Hedgehog signaling in NIH-3T3 cell lines, IC_50_ = 250 nM)	(43)
14. HiP-8	Hepatocyte growth factor (HGF) (Kd= 0.93 nM and IC_50_ = 0.9 nM)	*In vitro* (inhibition of HGF-induced activation of the MET in human mesothelioma, B16-F10 melanoma, and lung cancer cell lines); *in vivo* (inhibition of HGF in the PC-9 mouse xenograft model)	(44)
15. SUPR4B1w	microtubule-associate protein light chain (LC)3 (Kd= 120 nM for LC3A and 192 nM for LC3B)	*In vitro* (inhibition of autophagy in HeLa cells)	(45)
16. CM_11_-1	E3 ligase E6-associated protein (E6AP) (Kd= 0.6 nM)	*In vitro* (inhibition of ubiquitination of targets protein catalyzed by E6AP)	(46)
17. ATSP-7041	MDM2 and MDMX (Kd= 0.91 nM for MDM2 and 2.31 nM for MDMX)	*In vitro* (activation of p53 signaling in SJSA-1 and MCF-7 cell lines); *in vivo* (anti-tumor effect in SJSA-1 and MCF-7 mouse xenograft models)	(47)
18. ALRN-6924	MDM2 and MDMX (IC_50_ = 7.7 nM for MDM2 and IC_50_ = 24.7 nM for MDMX)	*In vitro* (activated p53-dependent transcription); *in vivo* (antitumor effect in a MOLM13 mouse xenograft model and eight TCL PDX models, activated anti-tumor immune response in Colon26 allografts, and synergistic effect in MCF-7 and ZR-75-1 mouse xenograft models); clinical trial (NCT02264613)	(48–52)
19. hD1	USP22 (Ki=180 nM and IC_50 =_ 100 nM)	*In vitro* (increased H2B ubiquitination in HEK293T cells)	(53)
20. Ub4a	Lys48-linked Ub chains (Kd=9± 3 nM)	*In vitro* (induction of apoptosis in U87, SH-SY5Y, MDA-MB-231, and HeLa cell lines); *in vivo* (antitumor effect in the human CAG myeloma cell mouse xenograft model)	(54)
21. mJ08-L8W	Lys48-linked Ub chains (Kd= 1.2 nM)	*In vitro* (induction of apoptosis in U87 cell lines)	(55)
22. KS-58	KRAS-G12D (EC_50 =_ 22 nM)	*In vivo* (anti-cancer activity in the PANC-1 mouse xenograft model)	(56)
23. MP-3995	KRAS (IC_50 =_ 0.5 nM)	*In vitro* (antiproliferative effect in eight KRAS mutant cell lines)	(57)

MM, multiple myeloma; NSCLC, non-small cell lung cancer; NHL, non-Hodgkin lymphoma; TCL, T- and NK-cell lymphomas; PDX, patient-derived xenografts.

## Macrocyclic peptides targeting extracellular PPIs

**Figure 1:**
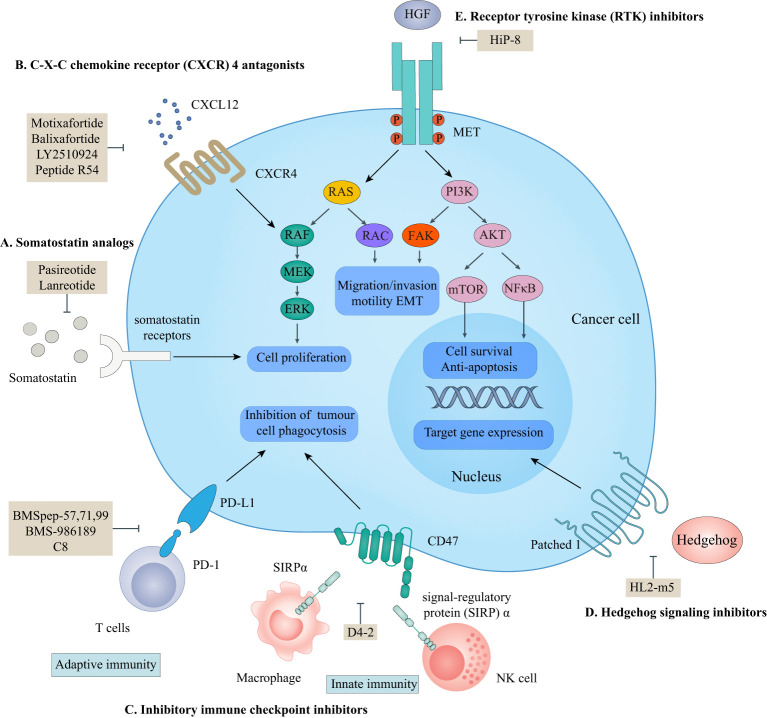
Macrocyclic peptides targeting extracellular protein-protein interactions. This scheme shows macrocyclic peptides-targeted extracellular proteins, the related signaling pathways, and their cellular functions. **(A)** somatostatin analogs. Lanreotide and pasireotide are somatostatin analogs. They can inhibit the activation of somatostatin receptors by endogenous somatostatin to inhibit cell proliferation. **(B)** C-X-C chemokine receptor (CXCR4) antagonists. Motixafortide, balixfortide, LY2510924, and Pep R54 can inhibit the interaction between CXCR4 and CXCL12, which is essential for cancer cell proliferation. **(C)** Inhibitory immune checkpoint inhibitors. BMSpep-57,77,99, BMS-986189, and C8 can inhibit the interaction between programmed cell death protein 1 (PD-1) and programmed cell death ligand 1 (PD-L1), which negatively modulate the adaptive immune systems. D4-2 can inhibit the interaction between CD47 and signal-regulatory protein (SIRP)α, which releases an inhibitory ‘do not eat me’ signal to lead to cancer cell evasion of immune detection and clearance. The PD-1/PD-L1 axis and CD47/SIRPα axis are critical for cancer immunotherapy. **(D)** Hedgehog (HH) signaling protein inhibitors. HL2-m5 can inhibit the activation of the HH pathway, which regulates target gene expression. **(E)** Receptor tyrosine kinase (RTK) inhibitors. HiP-8 can inhibit the hepatocyte growth factor (HGF)-mesenchymal-epithelial transition tyrosine kinase receptor (MET) interaction which is critical for cancer cell proliferation, migration, and invasion.

According to the ‘Rule of Five (Ro5)’, molecules with low molecular weight (MW below 500), the calculated logP below 5, fewer than five hydrogen bond donors, and fewer than ten hydrogen bond acceptors demonstrate good cell-permeability (6). Macrocyclic peptides violate all of the above parameters and lead to the fact that the unsatisfying cell permeability might be the Achilles’ heel of macrocyclic peptides. In this circumstance, targeting extracellular PPIs, especially the interactions between cell-surface receptors and their ligands, seems to provide an avenue for the clinical applications of macrocyclic peptides to bypass delivery challenges (**Figure 1**) (5, 58).

### Somatostatin analogs

**Figure 2:**
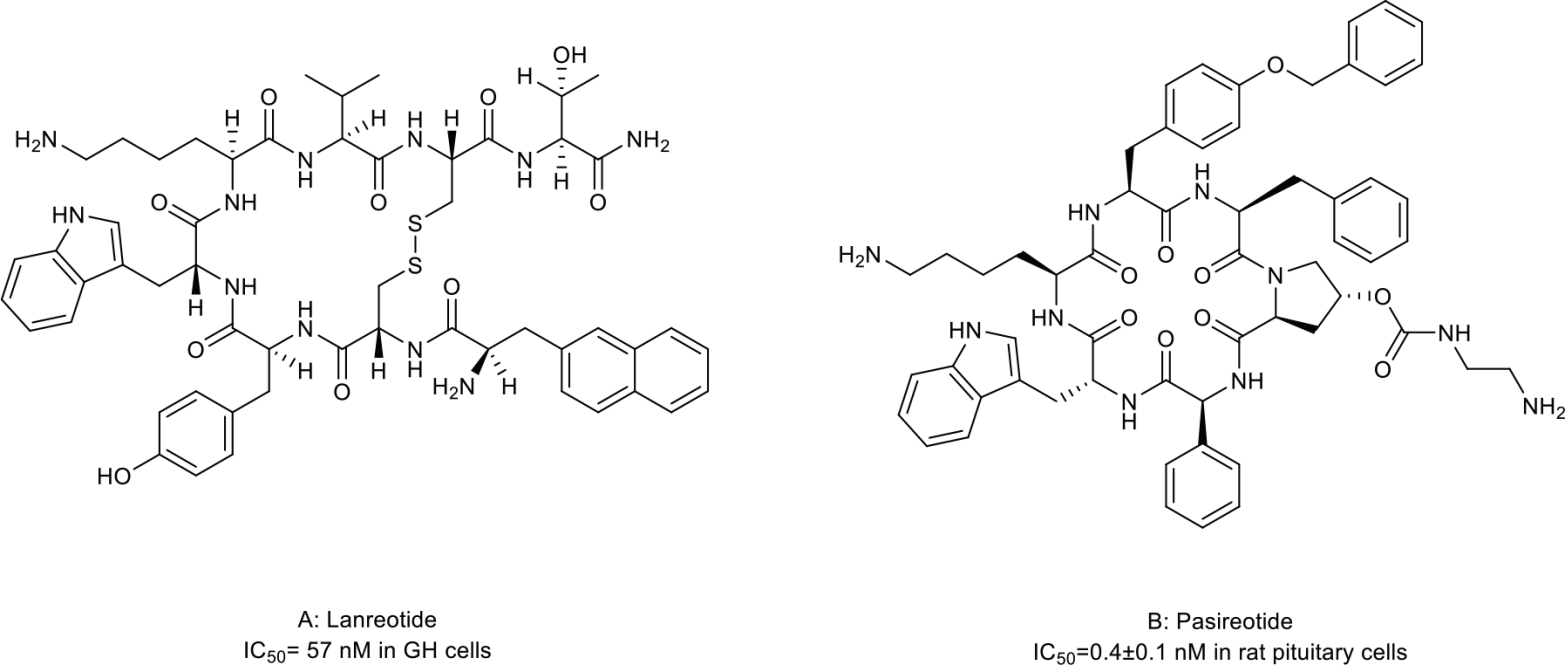
Macrocyclic peptides targeting somatostatin receptors. **(A)** Lanrenotide. IC50: 57 nM in GH cells; **(B)** Pasireotide, IC50: 0.4±0.1 nM in rat pituitary cells.

Somatostatins are a family of natural cyclic peptide hormones expressed in pancreatic δ-cells, the gastrointestinal tract, neuronal cells, and certain tumors. Somatostatins can activate somatostatin receptors (SSTRs) which culminate in the inhibition of hormonal secretion, modulation of neuronal ion channel transmission, and cell growth arrest (59–61). SSTRs are a family of G-protein-coupled receptors (GPCRs), which include five subtypes termed SSTR1-5. The SSTRs incidence varies among different tumor types. Neuroendocrine tumors and tumors of nervous systems express high densities of SSTR2. But other tumor types, such as renal cell carcinoma (RCC), lymphoma, and inactive adenoma, have less SSTR2 or other SSTR subtypes (62, 63). The rationale for the oncology therapeutic use of SSAs depends on the expression of SSTR subtypes in relevant tumor tissues. The activation of SSTRs induced by SSAs results in the inhibition of tumor-associated pathophysiological hormonal secretion and tumor growth (**Figure 1A**) (61). Two macrocyclic peptide drugs derived from somatostatin, lanreotide (**Table 1**; **Figure 2A**, compound 1) and pasireotide (**Table 1**; **Figure 2B**, compound2), have been approved by the FDA in clinical practice up to now.

Lanreotide, the first-generation SSA, is a cyclic octapeptide. It has a high affinity to SSTR2 and less affinity to SSTR3 and SSTR5. Pasireotide, the second-generation SSA, is a cyclic hexapeptide. It has the same affinity to SSTR2 but a higher affinity to SSTR1, 3, and 5 than lanreotide (64, 65). Both have been approved by the FDA to treat Cushing’s disease and acromegaly, which are characterized by chronic hypersecretion of hormones from a pituitary adenoma (**Table 2**) (14, 66, 67). In addition, lanreotide has been approved to treat neuroendocrine tumors. In addition, lanreotide has been approved to treat neuroendocrine tumors. It was reported that lanreotide inhibited the growth of the rat GH3 pituitary tumor cell line (IC_50_ = 57 nM) (18). And, pasireotide inhibited the growth hormone release in rat pituitary cell lines (IC_50_ = 0.4 ± 0.1 nM) (20). Multiple high-quality clinical trials and case series have demonstrated alleviated symptoms, a statistically significant increase in time to progression (TTP)/progression-free survival (PFS), long-term safety profile, and sustained antitumor effects upon treatment with lanreotide or pasireotide in patients with Cushing’s disease, acromegaly or neuroendocrine tumors (68–77).

On the other hand, SSAs may also have potential applications in non-endocrine tumor types because these tumors also express SSTRs. Thus, the off-label use of lanreotide or pasireotide to treat such diseases may also be promising. Despite the complete mechanisms of the antitumoral activity of SSAs not been demonstrated yet, some of the possible action mechanisms, including tyrosine kinase inhibition, induction of cell cycle arrest, proapoptotic effect, and inhibition of cancer cell adhesion and tumor angiogenesis, have been reported (78, 79). Lanreotide and pasireotide demonstrated antitumor effects in several preclinical models, including those for pancreatic ductal adenocarcinoma (PDAC), oral cavity squamous cell carcinoma, thyroid cancer, and prostate cancer (19, 21–26). However, the clinical data obtained from these non-endocrine tumors are still limited and discouraging. These two drugs showed no or limited benefit in several clinical cohorts (78, 80–83). The controversy between the preclinical and clinical data may result from uninvestigated SSTR statutes of enrolled patients and insufficient tumor cytotoxicity of SSAs. New generation SSAs with a better affinity to a broader range of SSTR subtypes and bigger tumor cytotoxicity and better planned clinical trials are needed to evaluate the role of SSAs in non-endocrine tumors.

### CXC chemokine receptor 4 (CXCR4) antagonists

CXCR4 is overexpressed in more than 20 human cancer types and correlated with advanced disease status and poor prognosis (84–86). The interaction between CXCR4 and its natural ligand CXCL12 (also known as stromal cell-derived factor-1α, SDF-1α) comprises a biological axis that is a well-validated PPI therapeutic target. It plays critical roles in mobilizing cancer cells and hematopoietic stem cells from the bone marrow to the peripheral blood and cancer progression, including the proliferation, invasion, and angiogenesis of cancer cells (87). Currently, the only marketed CXCR4 inhibitor is plerixafor (AMD 3100, a small-molecule drug) for stem cell mobilization in non-Hodgkin’s lymphoma (NHL) and multiple myeloma (MM) patients. Up to now, four macrocyclic peptide drugs serving as CXCR4 antagonists have been reported, and three of them are in clinical trials (**Table 2**; **Figure 1B**) (88, 89).

**Figure 3:**
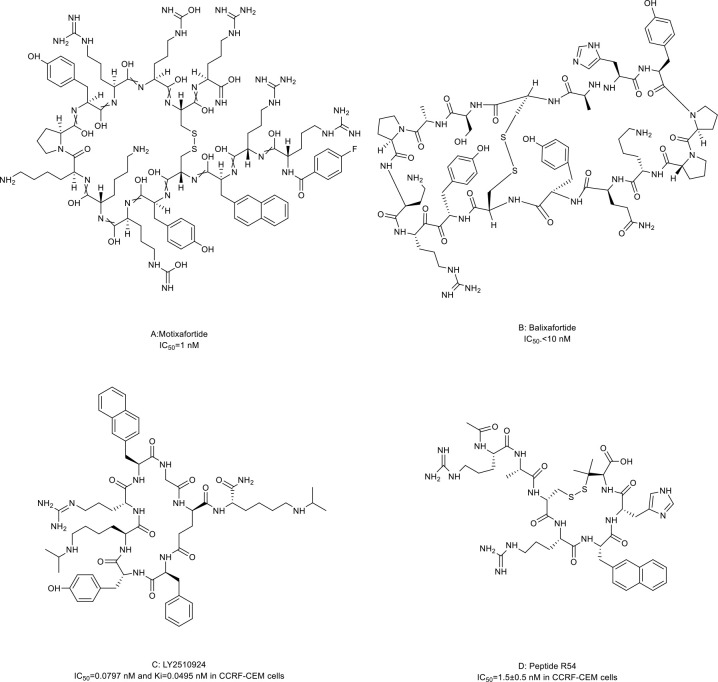
Macrocyclic peptides targeting CXC chemokine receptor 4 (CXCR4). **(A)** Motixafortide, IC50: 1 nM; **(B)** Balixafortide, IC50: 10 nM; **(C)** LY2510924, IC50: 0.0797 nM in CCRF-CEM cells; **(D)** Peptide R54, IC50: 1.5 nM in CCRF-CEM cells.

Motixafortide (BL-8040, BKT140) (**Table 2**; **Figure 3A**, compound 3), a heterodetic cyclic peptide, is the first peptide antagonist for CXCR4 and receives orphan drug designation for the treatment of pancreatic cancer from the European Commission and the FDA. It was modified from a natural protein named T22 (27, 90, 91). The inhibition mechanism of motixafortide is different from that of plerixafor. While plerixafor works as a weak partial agonist, motixafortide works as an inverse agonist with a higher affinity and a more lasting CXCR4 occupancy, showing an IC_50_ of 1 nM (28). It introduced unique cell-specific pro-apoptotic signaling pathways in MM and leukemia cells and induced apoptosis of acute myeloid leukemia (AML) blasts by altering the expression of miR-15a/16-1 (27, 29, 92). In addition, motixafortide may be an immune-modulatory agent by recruiting peripheral immune progenitor cells, which results in an enhanced antitumor immune response (93, 94). Both motixafortide monotherapy and combined therapy with chemotherapy or immunotherapy are safe with the profit from combination treatments in various cancer types (28, 30–34, 92). These cancers included AML, chronic myeloid leukemia (CML), MM, PDAC, and non-small cell lung cancer (NSCLC).

Balixafortide (POL6326) (**Table 2**; **Figure 3B**, compound 4) is also modified from T22 by the protein epitope mimetic approach (95). The ADME properties of balixafortide demonstrated a higher binding affinity (100-fold) and a prolonged binding to CXCR4 than plerixafor (96). Binding to CXCR4 with the β-hairpin mimicry, balixafortide inhibited the CXCL12-induced activation of downstream MAPK-ERK/PI3K-AKT pathways in the lymphoma and AML cell lines and blocked the CXCL12-dependent chemotaxis in breast cancer and leukemia cell lines (35, 95, 97). In addition, balixafortide can enhance the efficacy of chemotherapy by mobilizing AML cells that locate in a protective stromal microenvironment into circulation and lead to prolonged survival in the murine leukemia model (98). The profiles with excellent safety and tolerability of this drug have been observed in three early-phase clinical trials (36, 99, 100). Additionally, balixafortide plus eribulin showed potential anti-cancer activities among patients with HER2-negative metastatic breast cancer in a phase I clinical trial (NCT01837095) (36). Further evaluation of the comparative efficacy and safety of this combination versus eribulin monotherapy among patients with metastatic breast cancer in a randomized phase III trial (NCT03786094) is ongoing.

LY2510924 (**Table 2**; **Figure 3C**, compound 5) was developed by a medium throughput screen and rational design (37). It is a CXCR4 inhibitor featuring high *in vivo* stability but potentially with some safety issues and limited drug efficacy. Firstly, it showed reasonable preclinical activities in man tumor models. According to the structural modeling analysis, LY2510924 occupies the binding pocket in CXCR4 and possesses contacts with CXCR4 residues, including Arg30, Asp187, Arg188, Phe189, Gln200, His 113, Tyr190, and Glu288 (37). The potential mechanism of LY2510924 is that it may induce cell cycle arrest (G1 to G2-M progression) *via* inhibiting CXCL12-stimulated MAPK-ERK/PI3K-AKT and β-catenin pathways (37, 101). Monodrug therapy with LY2510924 demonstrated dose-dependent antitumor growth and anti-metastasis activities in leukemia, multiple solid tumor xenograft models, and a breast cancer metastatic model. When used in combination, it increased the efficiency of FLT3 inhibitors in preclinical FLT3-mutated AML models by suppressing TGF-β signaling (102). Secondly, the feature and advantage of LY2510924 is its high *in vivo* stability. The elimination half-life of LY2510924 is 9.16 hours (20 mg/day) in one phase I clinical trial, which was much higher than that of plerixafor (4.4-5.6 hours), motixafotide (0.29-0.72 hours) and balixafortide (5 hours) (28, 100, 103). Thus, a once-daily injection of LY2510924 for chronic treatment in clinical applications seems to be possible. However, the safety and clinical anti-cancer ability of LY2510924 need to be concerned. Serious adverse events occurred more frequently in the LY2510924 plus first-line standard of care (SOC) group than the SOC alone group (51% vs. 30.2%) in a small cell lung cancer (SCLC) phase II clinical trial (104). Additionally, the combination did not improve the drug efficacy over SOC monotherapy (median PFS: 5.88 months versus 5.85 months, p=0.98) (104). Similarly, LY2510924 also did not improve the drug efficacy when added to sunitinib in an RCC clinical trial (median PFS: 8.1 months versus 12.3 months) (105). Further investigation of LY2510924 to uncover the clinical mechanisms of LY2510924 is warranted.

Maro et al. (38, 106, 107) reported a novel macrocyclic peptide named Peptide R54 (Pep R54, **Figure 3D**, compound 6). According to the molecular dynamics results, the Arg4, 2-Nal5, and His6 side chains of Pep R54 occupied the minor and major pockets of CXCR4 (106). It mimiced CXCL12 to selectively bind the transmembrane bundle of CXCR4, which resulted in more efficient inhibition of CXCL12-mediated cell migration in a dose-dependent manner than plerixafor *in vitro*. In addition, Pep R54 displayed synergistic effects in combination with immunotherapy in a PES43 mouse xenograft model (38). These macrocyclic peptides of CXCR4 antagonists described above provide a valuable tool to target the CXCR4/CXCL12 axis in cancer therapy.

### Immune checkpoint inhibitors

Over the past decades, inhibitory immune checkpoint blockade has become the fifth pillar of cancer treatment beyond surgery, chemotherapy, radiation, and targeted therapy. Inhibitory immune checkpoints are critical for maintaining self-tolerance and minimizing collateral tissue damage when responding to pathogenic infection. This complex immune response relies on an interplay of both adaptive and innate immune systems (108, 109). However, these checkpoints may be engaged in cancer development and progression, leading to tumor immune surveillance escape and suppressed antitumor immune responses (110). Thus, increasing efforts have been paid to target inhibitory immune checkpoints with ICIs to enhance anti-cancer immunity.

The PD-1/PD-L1 axis is a well-established T cell immune checkpoint that negatively controls the adaptive immune systems (**Figure 1C**). PD-1 is predominantly expressed on the surface of antigen-stimulated T cells. Upon binding to PD-L1, which is frequently expressed on cancer cells and antigen-presenting cells, the PD-1/PD-L1 interaction reduces T cell activation, proliferation, and survival leading to T cell exhaustion and protecting cancer cells from destruction mediated by cytolytic T cells (3, 4).

**Figure 4:**
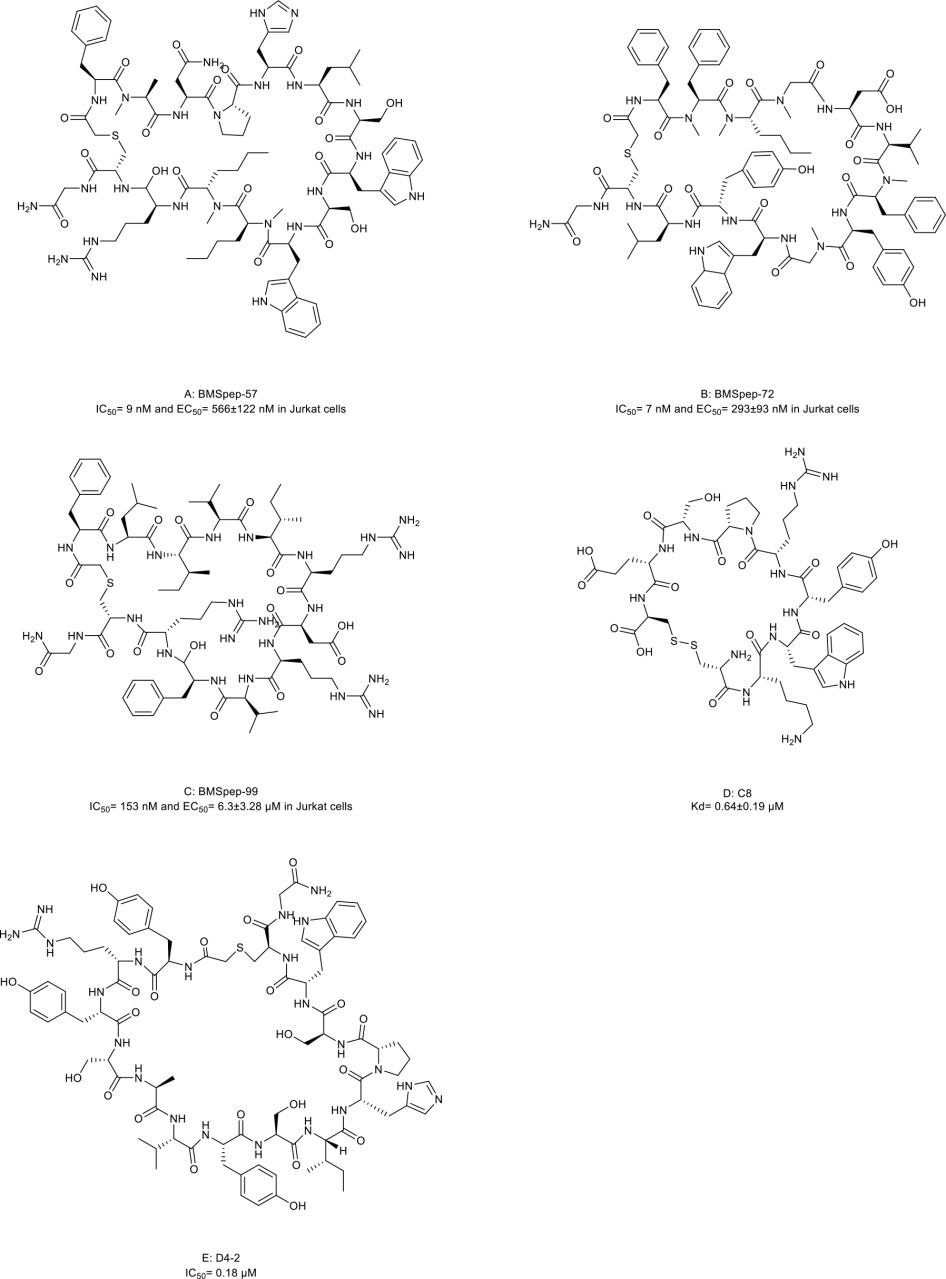
Macrocyclic peptides targeting immune checkpoints. **(A)** BMSpep-57, IC50=9nM and EC50 =566±122 nM in Jurkat cells; **(B)** BMSpep-71, IC50= 7nM and EC50 =293±93 nM in Jurkat cells; **(C)** BMSpep-99, IC50=153 nM and EC50 =6.3±3.28 µM in Jurkat cell; **(D)** C8, Kd=0.64±0.19 µM; **(E)** D4-2, IC50 = 0.18 µM

Several macrocyclic peptide inhibitors targeting the PD-1/PD-L1 axis have been developed. Bristol-Myers Squibb has reported two types of macrocyclic peptides for PD-1/PD-L1 blockade (**Figure 1C**) (111, 112). The first type of macrocyclic peptide includes BMSpep-57, BMSpep-71, and BMSpep-99 (**Figures 4A–C**, compound 7, 8, and 9). The IC_50_ values of these peptides were 9 nM, 7 nM, and 153 nM, respectively, in a homogeneous time-resolved fluorescence assay to determine their activities in inhibiting the interaction of PD-1/PD-L1 (39). These peptides restored the activities of the T-cell receptor-responsive promoter *in vitro* in a dose-dependent manner, and BMSpep-57 is the strongest one. The pharmacophore of these peptides is not related to other reported small-molecule PD-1/PD-L1 inhibitors, offering a blueprint for designing novel and more powerful antagonists of the PD-1/PD-L1 axis (113). The representative of another type of macrocyclic peptide developed by Bristol-Myers Squibb is BMS-986189 (compound 10), which completed the phase I clinical trial in 2018 (NCT0273973) in healthy people, but its structure was not released. BMS-986189 has a strong affinity for PD-L1 with an IC_50_ value of 1.03 nM, according to the related patents (40). Gao et al. (41) also reported a macrocyclic peptide named C8 (**Figure 4D**, compound 11) with high binding affinity with PD-1. The Arg5 and Cys9 of C8 form hydrogen bonds with the Thr76 and Asn 74 of the PD-1 to interfere with the PD-1/PD-L1 interaction. By interfering with the interaction and activating CD8^+^ T cells, C8 exerted antitumor effects in the CT26 mouse xenograft model in a CD8^+^ T cells-dependent manner.

On the other hand, due to the suboptimal response rates to ICIs targeting adaptive immune checkpoints, interest is growing in the innate immune checkpoints, especially the phagocytosis checkpoint (108). The signal-regulatory protein (SIRP)α-CD47 axis is the first identified tumor phagocytosis checkpoint. SIRPα, an inhibitory receptor, is expressed on myeloid cells such as macrophages and dendritic cells (DCs). It has an extracellular immunoglobulin (Ig)-like domain to bind its ligand CD47 which is often over-expressed on cancer cells. The interaction between CD47 and SIRPα transmits an inhibitory ‘do not eat me’ signal resulting in cancer cell evasion of immune detection and clearance (114–116). D4-2 is a macrocyclic peptide (**Figure 4E**, compound 12) containing 15 amino acids. It was developed as an allosteric inhibitor to target the Ig-like domain of SIRPα (**Figure 1C**) (42). It can tightly bind to SIRPα and form multiple intramolecular hydrogen bonds and salt bridges. D4-2 can not only enhance the antibody-dependent cellular phagocytosis activity of macrophages for antibody-opsonized cancer cells *in vitro* but also demonstrate a synergistic effect on tumor growth or metastasis in combination with rituximab and TA-99 *in vivo*. Besides, the preclinical safety profile of D4-2 is also encouraging. Only weak declines in total cholesterol and blood urea nitrogen were observed in immunocompetent mice when treated with D4-2.

### Hedgehog signaling protein inhibitors

**Figure 5:**
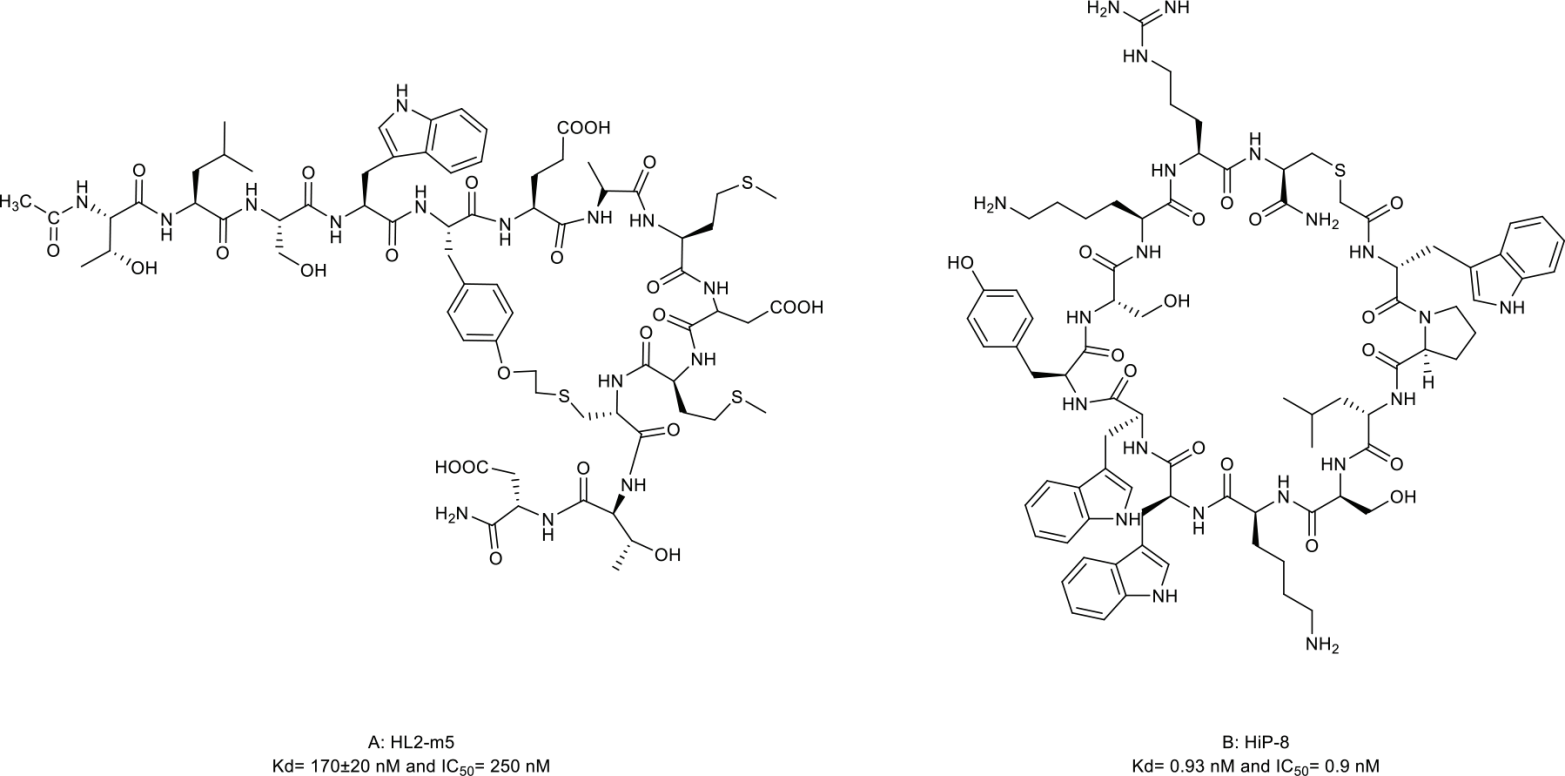
Macrocyclic peptides targeting Hedgehog (HH) signaling protein and receptor tyrosine kinases. **(A)** HL2-m5, Kdh170±20nM and IC50=290 nM; **(B)** HIP-8, Kd=0.93 nM and IC50=0.9 nM.

The HH signaling pathway plays a crucial role in embryonic patterning and development. However, the HH protein secreted by cancer cells induces the excessive activation of the HH signaling pathway in tumor-infiltrating stromal cells, which in turn contributes to the growth of cancer through several paracrine signals (117). Thus, the ligand-induced activation of the HH signaling pathway has emerged as one therapeutic target (**Figure 1D**). Owens et al. (43) identified a macrocyclic peptide named HL2-m5 (**Figure 5A**, compound 13) based on the HH protein-binding loop of the HH-interaction protein (**Figure 1D**). The Trp4 and Met10 residues of HL2-m5 formed potential interactions with the HH protein. HL2-m5 inhibited ligand-dependent HH signaling pathway activation and suppressed HH signaling-dependent gene transcriptions *in vitro*. In addition, the inhibitory activity of HL2-m5 is superior to that of robotnikinin, which is a small-molecule inhibitor (IC_50_: 250 nM vs. 15 μM).

### Receptor tyrosine kinase inhibitors

RTKs are a family of transmembrane receptors that play pivotal roles in regulating cell-to-cell communications and various cellular processes. Abnormal activation of RTKs leads to many types of human malignancies. Thus, RTKs have become important targets for therapeutic intervention, and RTK-based cancer therapies have reached widespread clinical use nowadays (118, 119). Macrocyclic peptides have been proposed to interfere with RTK activation by disrupting the ligand-receptor PPIs.

Mesenchymal-epithelial transition (MET) tyrosine kinase receptor is encoded by MET proto-oncogene. The abnormal activation of MET by its natural ligand hepatocyte growth factor (HGF) triggers a multistep signal transduction cascade involved in tumorigenesis and seems invariably correlated with poor prognosis (120–122). Thus, the oncogenic role of HGF/MET signaling has underpinned the clinical investigation of MET inhibitors. HiP-8 (**Figure 1E**, **5B**, compound 14), a macrocyclic peptide consisting of 12 amino acids, is identified by Katsuya et al. (44). It allosterically inhibited the HGF/MET interaction by interacting with the NK4 and SP domains of HGF in a dose-dependent manner, which, in turn, prevented MET activation *in vivo* (**Figure 1E**). In addition, when labeled with ^64^Cu, the HiP-8 variant served as a specific biomarker for noninvasive imaging of HGF-positive tumors using PET.

Other RTKs, such as epidermal growth factor receptor (EGFR), vascular endothelial growth factor receptor (VEGFR), and fibroblast growth factor receptor (FGFR), are also frequently over-expressed in many forms of human malignancies. They play critical roles in the malignant growth and the progression of solid tumors (123, 124). Yin et al. (125), Imanishi et al. (126), and Stanton et al. (127) reported several macrocyclic peptides against EGFR and VEGFR by inhibiting the ligand-receptor PPIs. Similarly, Lipok et al. (128) reported a macrocyclic peptide, an FGF-FGFR interaction antagonist, which can block FGF-induced cell proliferation by 40%. Although more studies on the efficacy of these compounds are required, these macrocyclic peptides provide promising tools to target RTKs in cancer therapy.

## Macrocyclic peptides targeting intracellular PPIs

**Figure 6:**
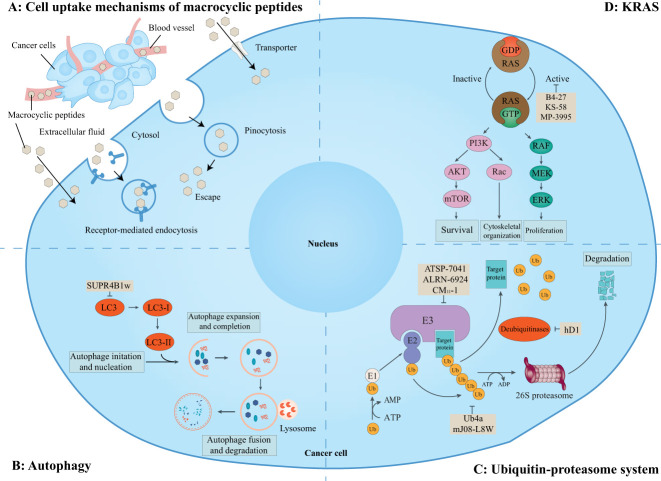
Macrocyclic peptides targeting intracellular protein-protein interactions. This schematic shows the cell uptake mechanisms of macrocyclic peptides, intracellular proteins, related signaling pathways, and cellular functions. **(A)** Overview of cell uptake mechanisms of macrocyclic peptides. Some macrocyclic peptides can cross the cell membrane in a passive way as small-molecule drugs. Other macrocyclic peptides may cross the cell membrane via receptor-mediated endocytosis, pinocytosis and pinosomal escape, and active transportation. **(B)** Macrocyclic peptides can target microtubule-associate protein light chain (LC)3 which is essential for the maturation of the autophagosome. **(C)** Macrocyclic peptides can target the ubiquitin-proteasome system which regulates many aspects of cell biology. **(D)** Macrocyclic peptides can target KRAS mutations which are important for cell survival, proliferation, and cytoskeletal organization.

It is estimated that there are about 130,000 binary interactions between human proteins and most of which are intracellular PPIs. These PPIs are generally undruggable to traditional small-molecule drugs. Thus, it has been pursued for many years to target these intracellular PPIs with macrocyclic peptides. Although violating the Ro5, some macrocyclic peptides can enter cells by passive diffusion, endocytosis and endosomal escape, direction translocation, or binding to membrane transporters (**Figure 6A**) (129, 130). Although the cellular activities of some of these cell-penetrating macrocyclic peptides have not been tested, macrocyclic peptides have emerged as promising modalities for regulating intracellular PPIs and are being exploited for drug discovery (**Figure 6**) (129).

This schematic shows the cell uptake mechanisms of macrocyclic peptides, intracellular proteins, related signaling pathways, and cellular functions. A) Overview of cell uptake mechanisms of macrocyclic peptides. Some macrocyclic peptides can passively cross the cell membrane as small-molecule drugs. Other macrocyclic peptides may cross the cell membrane *via* receptor-mediated endocytosis, pinocytosis and pinosomal escape, and active transportation. B) Macrocyclic peptides can target microtubule-associate protein light chain (LC3) which is essential for the maturation of the autophagosome. C) Macrocyclic peptides can target the ubiquitin-proteasome system which regulates many aspects of cell biology. D) Macrocyclic peptides can target KRAS mutations which are important for cell survival, proliferation, and cytoskeletal organization.

### Inhibitors of autophagy

Massive preclinical studies have suggested that the inhibition of autophagy may be a possible and powerful therapeutic strategy to improve outcomes in cancer patients (131, 132). Chloroquine and hydroxychloroquine are the only clinically available autophagy inhibitors (132, 133). However, the therapeutic windows of these two drugs are very narrow, and the toxicity caused by the drugs at therapeutically relevant doses illustrates the need to develop new drugs to target autophagy.

**Figure 7:**
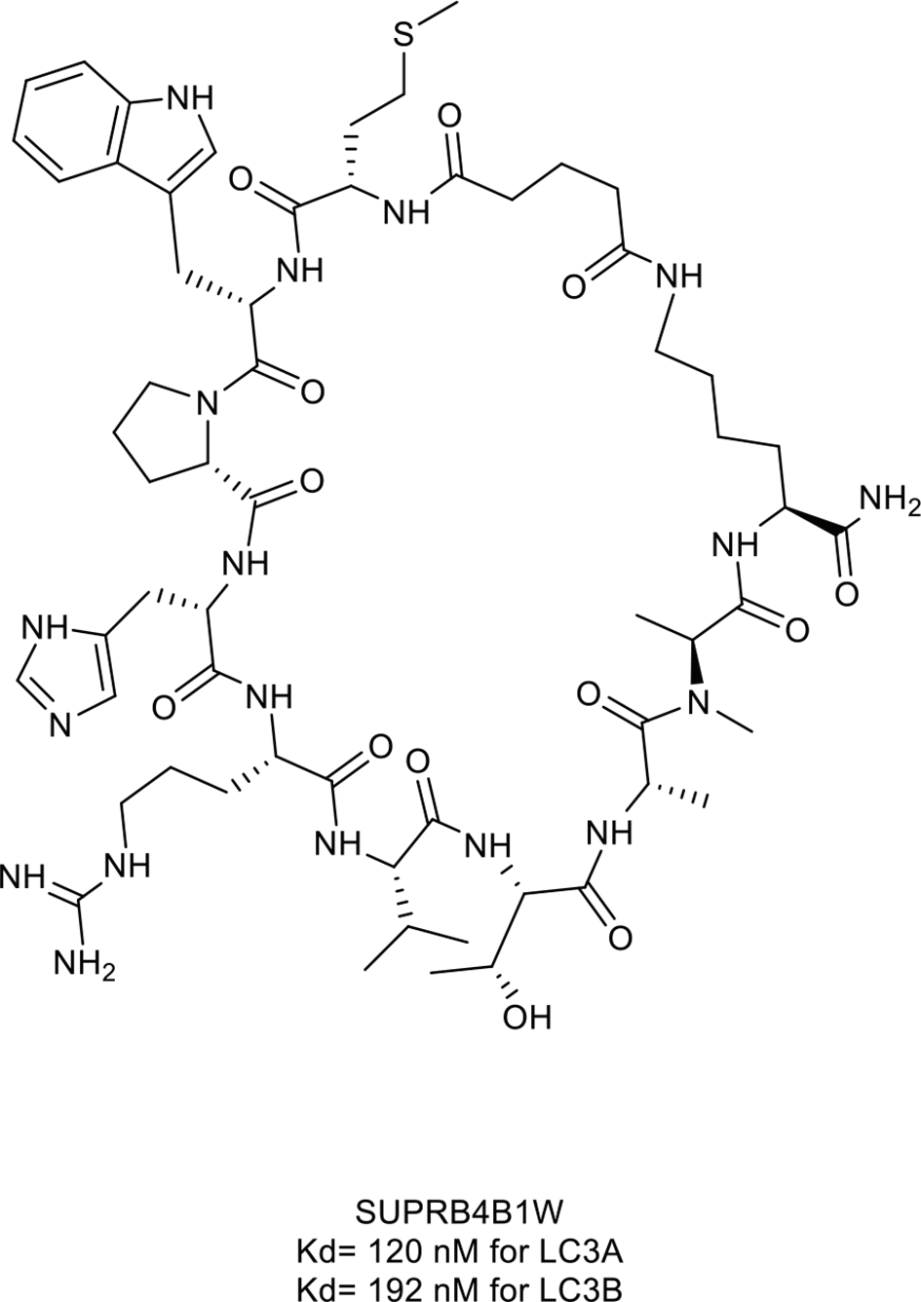
Macrocyclic peptide targeting autophagy. HL2-m5, Kd= 120 nM for LC3A and 192 nM for LC3B.

Gray et al. (45) identified a macrocyclic peptide named SUPR4B1w (**Figure 7**, compound 15), which targets microtubule-associate protein light chain (LC)3. LC3, as the core of the autophagy process, is essential for the maturation of the autophagosome (134). The Trp1 and Val5 residues of SUPR4B1w interacted with LC3 and further blocked autophagosome maturation in a dose-dependent manner and re-sensitized several resistant cell lines to cisplatin-mediated cytotoxicity (**Figure 6B**). Furthermore, the combination of SUPR4B1w with carboplatin induced almost complete inhibition of intraperitoneal tumor outgrowth in a mouse model of metastatic cancer.

### Ubiquitin-proteasome system

The UPS plays important roles in many aspects of cell biological processes by marking proteins with ubiquitin followed by degradation of target proteins. The core of ubiquitination is the modification of protein substrates by ubiquitin (Ub) or polyUb chains. This process is precisely regulated by both the sequential interaction of ubiquitin-activating enzymes (E1s), ubiquitin-conjugating enzymes (E2s) and ubiquitin ligases (E3s) and deubiquitinating enzymes (DUBs) (135, 136). E3 ligases, as the key components at the last step of the ubiquitination cascade, are responsible for transferring ubiquitin to substrates and Ub chain topology. DUBs, conversely, cleave Ub from Ub chains to reverse the ubiquitination. Dysregulation of the UPS is associated with carcinogenesis, invasion, and cancer cell proliferation. Thus, targeting the UPS has been an attractive target for cancer therapy (**Figure 6C**) (137–139).

#### Inhibitors for E3 ubiquitin ligases

The homeostasis of the transcription factor p53, a famous tumor suppressor, is critical for its tumor-suppressive function. The inactivation of p53 is a hallmark of virtually all cancers (140). Ubiquitination is one key regulator of p53 stability (141).

**Figure 8:**
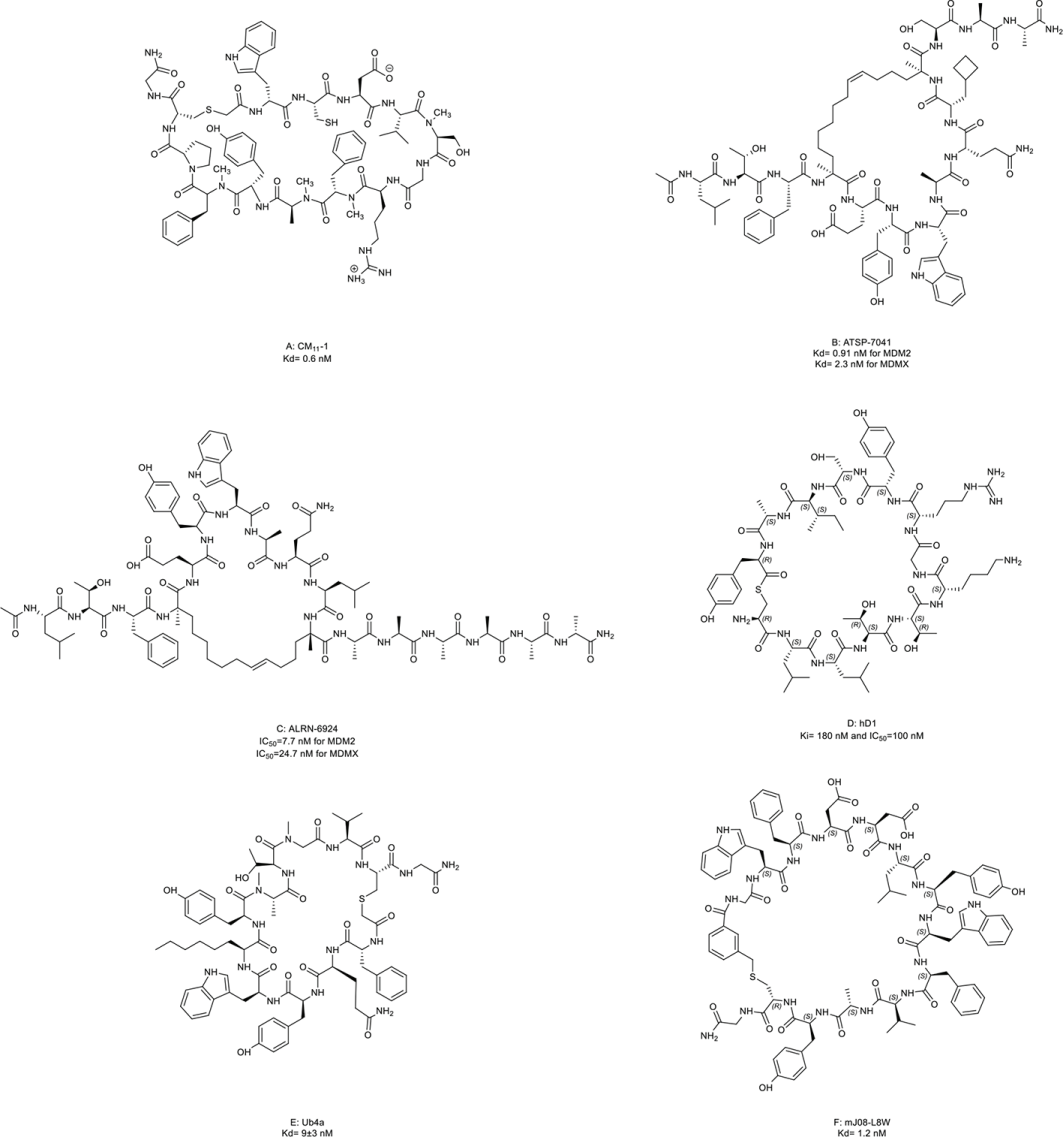
Macrocyclic peptides targeting the ubiquitin-proteasome system (UPS). **(A)** CM11-1, Kd= 0.6 nM; **(B)** ATSP-7041, Kd= 0.91 nM for MDM2 and 2.3 nM for MDMX; **(C)** ALRN-6924, IC50=7.7 nM for MDM2 and 24.7 nM for MDMX; **(D)** hD1, Ki= 180 nM and IC50=100 nM; **(E)** Ub4a, Kd= 9±3 nM; **(F)** mJ08-L8W, Kd= 1.2 nM.

In epithelial tumors induced by human papillomaviruses (HPV), p53 is recruited and degraded by the HPV oncoprotein E6 and E3 ligase E6-associated protein (E6AP) (142). Yamagishi et al. (46) identified an anti-E6AP macrocyclic peptide inhibitor named CM_11_-1 (**Figure 8A**, compound 16) that can prevent the E6AP-catalyzing polyubiquitination on p53 *in vitro*.

Later, an E3 ligase MDM2 and its homolog MDMX were found to degrade p53 without exogenous factors. The heterodimerization of MDM2 with MDMX plays a crucial role in p53 inhibition contributing to cancer progression (2, 143). Aileron Therapeutics developed two stapled α-helical peptides named ATSP-7041 (**Figure 8B**, compound 17) and ALRN-6924 (**Figure 8C**, compound 18) as dual inhibitors of MDM2 and MDMX. These two macrocyclic peptides mimiced the transactivation domain of p53 with an α-helical to bind MDM2 and MDMX. ATSP-7041 interacted with the MDMX binding pocket through Van der Waals contacts, hydrogen bonds, and a cation-π interaction (47). ATSP-7041 reactivated p53 by disrupting p53/MDM2 and p53/MDMX complexes, leading to suppressed proliferation in multiple cancer cell lines *in vivo* (47, 144–146). ALRN-6924 is the advanced analog of ATSP-7041. The investigational new drug (IND) application of ALRN-6924 has been accepted by the FDA as a myelopreservation agent in patients with p53-mutant cancer who received chemotherapy. Similarly, the potent *in vivo* and *in vitro* anti-cancer effects of ALRN-6924 through the dose- and time-dependent dual inhibition of MDM2/MDMX have been observed in various preclinical models (48, 49, 147). In addition, ALRN-692 overcame resistance to ICI therapy *in vivo* and improved the antitumor efficacy of chemotherapy in breast cancer models (50, 51). The data from the phase I clinical trial (NCT02264613) of ALRN-6924 in 71 patients with solid tumors and lymphomas showed that ALRN-6924 was well-tolerated and demonstrated promising antitumor activity (52). The disease control rate was 59%, including two confirmed complete response cases and two confirmed partial responses. In addition, Li et al. (148) and Sang et al. (149) also reported several macrocyclic peptides targeting MDM2/MDMX.

#### Inhibitors of deubiquitinase or Ub chains

USP22 is a deubiquitinase that removes ubiquitin from histone 2B and serves as an oncogenic driver (150). Macrocyclic peptide inhibitors of USP22 have been developed by Morgan et al. (53) with one representative named hD1 (**Figure 8D**, compound 19). It can selectively inhibit USP22 *in vivo*.

Some macrocyclic peptides specifically target Lys48-linked Ub chains, which are critical in inducing target protein degradation by the 26s proteasome (54, 55, 151, 152). Ub4a (**Figure 8E**, compound 20) and mJ08-L8W (**Figure 8F**, compound 21) are two representatives of these compounds. By binding to the Lys48-linked Ub chains and Lys48-linked ubiquitin dimers, they disrupted the recognition of proteasome in a dose-dependent manner which further resulted in the apoptosis of tumor cells *in vitro* and inhibited tumor growth *in vivo*.

### KRAS inhibitors

The Rat sarcoma (RAS) family of proto-oncogenes are the most frequently mutated oncogenes observed in about 30% of cancers. The protein members of this family include HRAS, NRAS, and KRAS. KRAS is the most commonly mutated isoform in cancers. When binding to GTP, small GTPases encoded by RAS are converted into active forms to stimulate downstream signaling pathways and regulate various cell functions. Mutated small GTPases encoded by oncogenic RAS are locked in an active state, thereby constitutively triggering downstream oncogenic pathways (153, 154). Due to a lack of suitable surface pockets for small-molecule inhibitors, RAS has once been considered an undruggable target (155). In 2021, based on several encouraging results from clinical trials, sotorasib, a small-molecule inhibitor, resulted in the FDA accelerated approval as the first KRAS-targeted therapy (156). In 2022, the FDA accepted the New Drug Application for adagrasib, another small-molecule inhibitor, for treating patients with NSCLC harboring the KRAS G12C mutation (157). Although progress has been made, targeting KRAS mutations remains a significant challenge in drug development.

Macrocyclic peptides against RAS have been shown as promising strategies to treat such RAS mutant cancers (**Figure 6D**). Buyanova et al. (158) reported a pan-RAS inhibitor named B4-27, a bicyclic peptide. It blocked the interaction between activated RAS and effector proteins, leading to the apoptosis of RAS-mutant cancer cells and suppressed tumor growth *in vivo* at low doses (≤5 mg/kg). However, pan-RAS inhibition may not be applicable in clinical because wild-type RAS is essential in normal cell signaling and may create toxicity concerns. In addition, different mutations in KRAS have distinct biochemical properties which may influence the therapeutic response (159). Thus, inhibitors against these specific mutants are being developed for RAS-targeted therapy.

**Figure 9:**
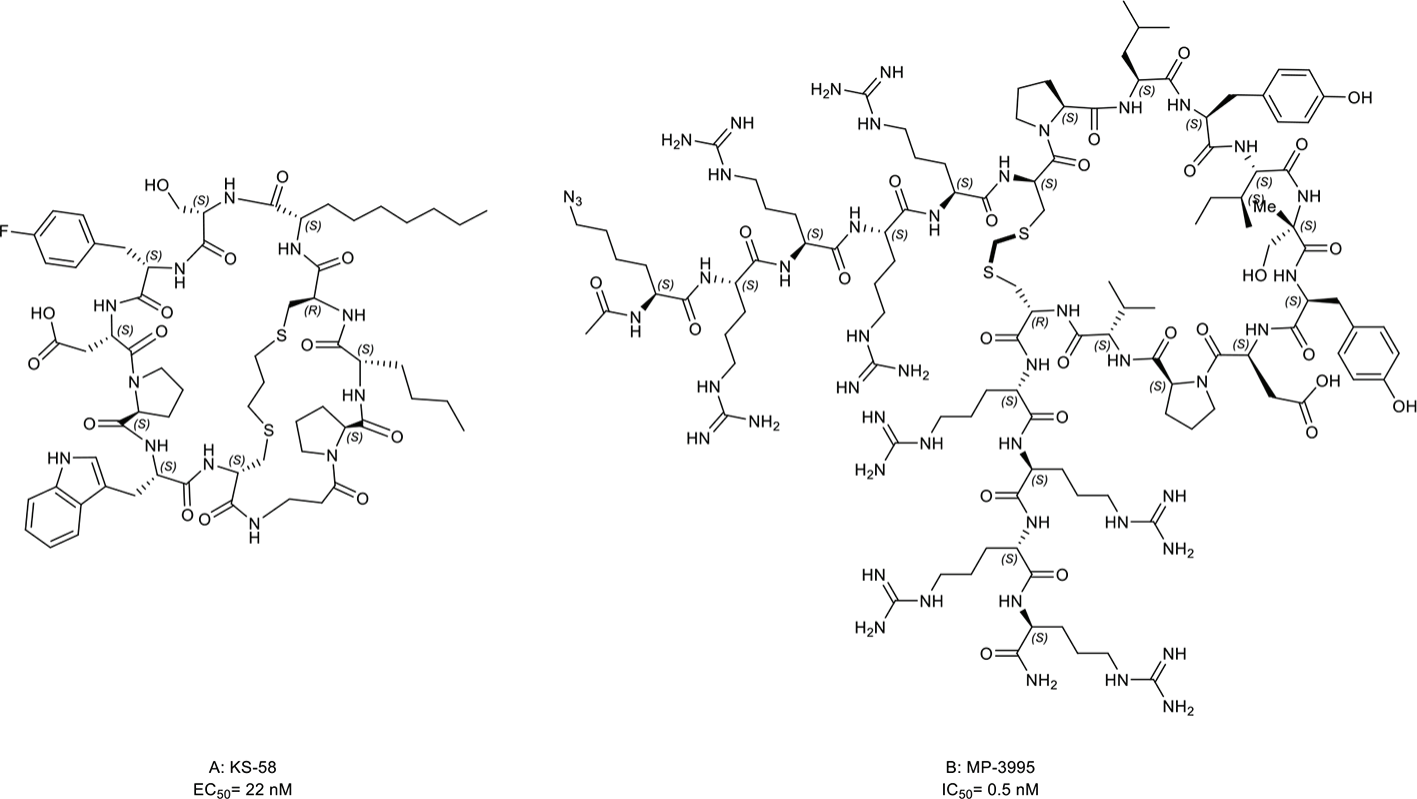
Macrocyclic peptides targeting KRAS. **(A)** KS-58, EC50= 22 nM; **(B)** MP-3995, IC50= 0.5 nM.

KRpep-2d, a macrocyclic peptide, is the first reported selective KRAS-G12D inhibitor (160, 161). Although this compound has the disadvantages of low cellular activity and induction of mast cell degranulation, it provides a solid basis for the subsequent development of KRAS inhibitors (57). Based on its scaffold, two macrocyclic peptides, KS-58 (**Figure 9A**, compound 22) and MP-3995 (**Figure 9B**, compound 23) were developed with higher binding affinity, increased cell permeability, and better cellular activity. KS-58 is the first KRAS-G12D inhibitor with anti-cancer activity *in vivo* reported by Sakamoto et al. (56). KS-58 formed hydrophobic and cation–π stacking interactions with KRAS. In addition, KS-58 had synergistic growth inhibitory effects in the PANC-1 mouse xenograft model when combined with the drug gemcitabine. However, KS-58 required a high dose to show its efficacy despite no adverse side effects observe. Later, Lim et al. (57) reported another improved peptide named MP-3995 with anti-cancer activity *in vitro*. The favorable attribute is that MP-3995 only shows the antiproliferative effect in KRAS dependent cancer cell lines but has no effect in KRAS independent cell lines, displaying its high selectivity and potential lower toxicity. These compounds are still in their infancy but are likely to enter a clinical trial for KRAS mutant tumors. Additionally, Zhang et al. (162) reported three new macrocyclic peptides scaffold targeting the KRAS-G12D mutation, which was different from KRpep-2d. Cell-permeable macrocyclic peptides for KRAS-G12V mutation have also been developed by Pei et al. which blocked KRAS activated signaling pathway and induced apoptosis of cancer cells *in vitro* (163, 164). They may begin a new chapter in the discovery of KRAS inhibitors.

## Discussions

Molecules following the ‘Rule of Five (Ro5)’ are proposed to demonstrate good cell permeability (6). However, macrocyclic peptides seem to violate all five parameters to be perfect drug candidates because their molecular weights are higher than 500 (**Table 3**) and because they have more hydrogen bond donors and acceptors in their structures (**Figures 2**–**5**, **7**–**9**). But this does not prevent them from being successful drugs. First, the cell permeability of macrocyclic peptides was moderate between the high permeability of small molecules and the low cell permeabilities of acyclic peptides (**Table 3**). Second, many macrocyclic peptides have shown high efficacy *in vivo* in mouse models and clinical trials. Third, the facts have proved that macrocyclic peptides can serve as successful drug candidates. The FDA has now approved many macrocyclic peptide drugs to treat human diseases, including cancer (**Table 1**). The successful use of these drugs in disease treatment has stimulated increasing attention to developing more macrocyclic peptide drugs.

The field of macrocyclic peptides is currently at an exciting stage. Macrocyclic peptides have displayed functional diversification and broad signaling plasticity. Targeting dysregulated PPIs with macrocyclic peptides is a promising strategy in cancer therapy. According to the statistics from the website of the National Cancer Institute, cancer is one of the leading causes of death worldwide, and each year cancer incidence increases gradually. The number of new cancer cases is expected to rise to 29.5 million by 2040. Additionally, the annual cancer-related cost is also huge. For example, the estimated national expenditures for cancer care in the united states in 2018 were $150.8 billion. And in the future, cancer costs are likely to elevate due to the increase in cancer incidence. Therefore, the sales of cancer drugs, including macrocyclic peptide drugs, are huge. Meanwhile, the estimated overall expense of peptide drugs might be lower than that of small-molecule drugs with the progression in synthetic methodology. Thus, macrocyclic peptide drugs will provide a more cost-effective way to treat cancer patients.

However, it remains premature to judge whether macrocyclic peptides are more favored over small-molecule drugs or biologics in all cases. There are still some challenges that need to be addressed.

First, membrane permeability remains one of the most critical challenges. Key advances in developing highly active cell-penetrating peptides have been achieved in recent decades, such as amino acid substitution and modifications of the peptide backbone. Some of these studies have been well-reviewed recently (129, 165). With the progress on new synthetic methodologies, for example, computer-aided design strategy, MOrPH-PhD, engineered tRNA, and ‘catch–release’ strategy, this obstacle may be conquered shortly (126, 166–172). In the meanwhile, studies on the pharmacokinetics of macrocyclic peptides, especially the tissue selectivity, elimination mechanism, and interaction between macrocyclic peptides and concomitant medications, are rare. Given that macrocyclic peptides are not identical to small-molecule drugs and biologics as drugs, a good understanding of these factors is critical for drug development timelines, which will influence the success probabilities for these novel compounds. Further research on the pharmacodynamics of macrocyclic peptides is required to warrant a better understanding of macrocyclic peptides.

Second, although some macrocyclic peptides have demonstrated excellent pre-clinical anti-cancer activities, the efficacy does not always successfully translate into the clinic. The most representative case is cilengitide, developed by Merck. Cilengitide, an αvβ3 and αvβ5 integrin inhibitor, is a macrocyclic RGD-containing peptide. In several early-phase clinical trials, cilengitide has demonstrated potential antitumor activity with improved survival in glioblastoma patients. However, the multicenter randomized phase 3 trial (NCT00689221) showed that cilengitide therapy did not improve the progression-free survival (PFS) and overall survival in glioblastoma patients. And cilengitide was announced as not being developed as an anti-cancer drug in the future (173). There were some possible reasons accounting for this. The first reason may lie in the inherent nature of the macrocyclic peptides. The poor membrane permeability, off-target effects, and insufficient bioactivity may contribute to the failure. Second, most proof-of-concept studies of macrocyclic peptides were conducted in cancer cell lines and animal models. Although animal disease models can simulate some aspects of cancer, these models cannot fully model human cancers’ complexity. Most pre-clinical studies used murine models for the activity and pharmacokinetics studies, which are different from human patients. In the future, more tools will be necessary to model cancer to validate potential drug candidates more reliably. Third, few reliable pre-clinical biomarkers are suitable to predict the clinical benefit of macrocyclic peptide drugs. Forth, comorbidities in patients with cancer can influence clinical trial decisions, which may lead to bias when conducting clinical trials (174). At last, the future design of clinical trials should base on pre-clinical research, biomarker status of patients, and clinical knowledge. It is puzzling that the tumor type can significantly impact drug response. The antitumor effects of SSAs were only observed in patients with a few specific tumors. It contradicts the fact that therapeutic targets broadly exist in various tumors. Future research directions can also focus on improving the anti-cancer efficacy of these drugs in other cancer types to expand their clinical indications. On the other hand, due to the heterogeneity of cancers, it is crucial to design efficient combination therapies in cancer treatment (175). However, the combination therapies of macrocyclic peptides with either chemotherapy or immunotherapy do not always show a profit. And, sometimes, they even increase toxicity. To further improve the success rate of these peptides in cancer treatment, it would be necessary to develop suitable combination therapies with other forms of therapy, such as chemotherapy, radiotherapy, or immunotherapy.

In summary, the boundary of oncology macrocyclic peptide drugs is expanding. While some challenges exist in the field, macrocyclic peptides still provide a unique opportunity to treat cancer. Macrocyclic peptides can be a weapon in our arsenal of anti-cancer therapeutics.

## Author contributions

XS, DG and BJ generated the concept. JY and XS wrote the draft, made the tables & figures. XS and JY revised the manuscript. HL helped to generate the chemical structures. All authors contributed to the article and approved the submitted version.

## Funding

The research was supported by the National Natural Science Foundation of China (Grant No. 82072592 to XS; No. 81972666 to DG), Shanghai Science and Technology Innovation Fund (Grant No. 20S11903100 to XS) and Shanghai Frontiers Science Center for Biomacromolecules and Precision Medicine at ShanghaiTech University to BJ.

## Acknowledgments

The authors acknowledge the useful discussions given by the members of Jiang lab in ShanghaiTech University. Thanks to Yu Hua from Shanghai Ninth People’s Hospital, Shanghai Jiao Tong University School of Medicine, for her kind support. Thanks to Yulin Cheng from Ruijin Hospital, Shanghai Jiao Tong University School of Medicine, and Chenyou Zhu from Tsinghua University for their kind help to check the chemical structures. Thanks to Qinzhou Lan from Zhongshan Hospital.

## Conflict of interest

The authors declare that the research was conducted in the absence of any commercial or financial relationships that could be construed as a potential conflict of interest.

## Publisher’s note

All claims expressed in this article are solely those of the authors and do not necessarily represent those of their affiliated organizations, or those of the publisher, the editors and the reviewers. Any product that may be evaluated in this article, or claim that may be made by its manufacturer, is not guaranteed or endorsed by the publisher.

## Glossary

**Table:**

AML	acute myeloid leukemia
CML	chronic myeloid leukemia
CXCR4	CXC chemokine receptor 4
DCs	dendritic cells
DUBs	deubiquitinating enzymes
E6AP	E6-associated protein
EGFR	epidermal growth factor receptor
FDA	US Food and Drug Administration
FGFR	fibroblast growth factor receptor
GPCRs	G-protein-coupled receptors
HGF	hepatocyte growth factor
HH	hedgehog
HPV	human papilloma viruses
IC_50_	half maximal inhibitory concentration
ICIs	immune checkpoint inhibitors
Ig	immunoglobulin
LC	microtubule-associate protein light chain
MDM2	mouse double minute 2
MET	mesenchymal-epithelial transition
MM	multiple myeloma
NHL	non-Hodgki's lymphoma
NSCLC	non-small cell lung cancer
PD-1	programmed cell death protein 1
PD-L1	programmed cell death ligand 1
PDAC	pancreatic ductal adenocarcinoma
PFS	progression-free survival
PPIs	protein-protein interactions
PTCL	cutaneous T-cell lymphoma
RAS	Rat sarcoma
Ro5	Rule of Five
RTKs	receptor tyrosine kinases
SCLC	small cell lung cancer
SDF-1α	stromal cell-derived factor-1α
SIRPα	signal-regulatory protein &alpha;
SOC	standard of care
SSTRs	somatostatin receptors
TTP	time to progression
Ub	ubiquitin
UPS	ubiquitin-proteasome system
VEGFR	vascular endothelial growth factor receptor
